# MSeq-CNV: accurate detection of Copy Number Variation from Sequencing of Multiple samples

**DOI:** 10.1038/s41598-018-22323-8

**Published:** 2018-03-05

**Authors:** Seyed Amir Malekpour, Hamid Pezeshk, Mehdi Sadeghi

**Affiliations:** 1 School of Mathematics, Statistics and Computer Science, College of Science, University of Tehran, Tehran, Iran; 2 School of Biological Sciences, Institute for Research in Fundamental Sciences, Tehran, Iran; 3 National Institute of Genetic Engineering and Biotechnology, Tehran, Iran; 4 Present Address: Department of Mathematics and Statistics, Concordia University, Montreal, Canada

## Abstract

Currently a few tools are capable of detecting genome-wide Copy Number Variations (CNVs) based on sequencing of multiple samples. Although aberrations in mate pair insertion sizes provide additional hints for the CNV detection based on multiple samples, the majority of the current tools rely only on the depth of coverage. Here, we propose a new algorithm (MSeq-CNV) which allows detecting common CNVs across multiple samples. MSeq-CNV applies a mixture density for modeling aberrations in depth of coverage and abnormalities in the mate pair insertion sizes. Each component in this mixture density applies a Binomial distribution for modeling the number of mate pairs with aberration in the insertion size and also a Poisson distribution for emitting the read counts, in each genomic position. MSeq-CNV is applied on simulated data and also on real data of six HapMap individuals with high-coverage sequencing, in 1000 Genomes Project. These individuals include a CEU trio of European ancestry and a YRI trio of Nigerian ethnicity. Ancestry of these individuals is studied by clustering the identified CNVs. MSeq-CNV is also applied for detecting CNVs in two samples with low-coverage sequencing in 1000 Genomes Project and six samples form the Simons Genome Diversity Project.

## Introduction

Copy Number Variation (CNV) and balanced rearrangements such as inversions and translocations are types of the large structural variations in the human genome and other organisms. In Copy Number Variation, a gene or a genomic region appears in different number of copies in different individuals or even in different cells of the same individual. CNVs are generally referred to as a duplication or deletion of a genomic region with at least 1 kb in length. However, several clinically important CNVs are shorter than 1 kb in length. CNV results in having variations in the gene expressions and abnormalities in the human phenotypes^1^. Moreover, CNV is envisaged to be associated with many human diseases such as autoimmune disease^2^, autism^1^ and developmental disabilities^3^, diabetes, schizophrenia^4^, cancer^3^ and obesity.

In the last decade, CNVs are studied via Microarray-based Comparative Genomic Hybridization (aCGH) methods^5–10^. However, the current aCGH platforms which benefit of more than 1 million genomic probes have a lower detection limit of CNVs of length ~5 kb to 25 kb^11,12^. In the recent years, Next Generation Sequencing (NGS) has provided new opportunities for the CNV studies with an unprecedented resolution^13–15^. In NGS, millions of single end or mate pair reads are generated from the sample genomes with shotgun sequencing. CNVs are then detected based on the frequency of the reads (read depth) or aberrations in the mate pairs, after mapping the short reads to the reference genome.

The majority of the current CNV detection tools analyze only one sample genome, at a time. These tools which are not capable of the simultaneous analysis of multiple samples rely either on read depth data e.g. CNV-seq^14^, rSW-seq^16^, m-HMM^17^, BIC-seq^18^, EWT^19^, SegSeq^20^, CNVwire^21^ and ReadDepth^22^ or on mate pair/split reads^23–34^. However, there are benefits in having the capability to analyze several sequencing samples, simultaneously.

Multiple sequencing reduces the effect of the systematic errors and artifacts which are attributed to the library-preparation protocol or individual sample genome characteristics^35^. There are common CNVs which are shared by complex diseases^36^ and can be detected from sequencing of multiple samples. Simultaneous analysis of multiple samples allows detecting read counts variations occurring due to the noise across samples, even in genomic positions with constant copy numbers.

Therefore, to increase the detection power, more samples should be sequenced with a low sequencing coverage rather than for sequencing a few samples with high-coverage sequencing. Indeed, CNV detection methods which rely on a low-coverage sequencing data are more relevant in the future studies^37,38^. Currently, many individuals are sequenced with a low genome-wide coverage. For example, the 1000 genomes project carried out the whole-genome shotgun sequencing of 179 individuals with 2× to 4× coverages^39–41^.

Currently, there are a few tools which are capable of the simultaneous analysis of several sequenced samples^37,42,43^. However, a major drawback of these tools is relying only on read depth data which results in suffering from a low power or a high false positive rate, due to the large noise in read depth signals. Moreover, these tools do not take the observed aberrations in the mate pair reads into account. Indeed, besides read depth, mate pair insertion sizes provide another source of information for the genome-wide CNV detection with an increased resolution.

In this paper, MSeq-CNV is proposed for detecting recurrent genomic deletions and duplications across multiple individuals, by the simultaneous analysis of samples. To the best of our knowledge, MSeq-CNV is the first computational tool which takes both read depth and insertion size signals in several individuals into account. The MSeq-CNV applies a mixture density to model the distribution of the read counts and the distribution of the number of mate pairs with aberrations in insertion size.

Each component in the mixture density applies a Binomial distribution to model the number of mate pairs with insertion size aberrations and a Poisson distribution to model the read counts, in each genomic region. After estimating the model parameters based on Expectation-Maximization (EM) algorithm, the posterior probability of the digitized copy number of each segment in the sample genomes is computed. The resolution of the MSeq-CNV is evaluated on a set of samples with implanted CNVs, which are constructed based on the human reference genome. Compared to the other state of the art methods, MSeq-CNV has reached an unprecedented precision and recall values which allows detecting recurrent genomic variants, accurately.

The MSeq-CNV is also applied for the CNV detection in a set of six HapMap individuals with high-coverage sequencing, including a CEU trio of European ancestry (NA12891, NA12892, and NA12878) and a YRI trio of Yoruba Nigerian ethnicity (NA19238, NA19239, and NA19240).

## Methods

Assume that there are k sample genomes which are sequenced using a Next Generation Sequencing platform and mate pair reads are generated. After mapping mate pairs, the reference genome is divided into T segments of length L. Here, we aim at estimating the copy number of each genomic segment in samples 1 to k. To estimate the copy number of each sample in the t^th^ genomic segment where t = 1, 2, …, T, this paper relies on two signals: i) number of reads which are mapped to the segment, and ii) information from the mate pair whose insertion (un-sequenced) region is passing the t^th^ genomic segment and its reads are flanking the corresponding genomic segment.

Here, number of reads which are generated from the j^th^ sample and are mapped to the t^th^ segment of reference genome (studied segment) is denoted by ${f}_{j}$. Also, ${n}_{j}$ denotes the number of mate pairs which are generated from the j^th^ sample and their insertion region is passing the t^th^ segment, after mapping to it.

### Signal characteristics in different genomic states

The studied segment in the j^th^ sample has one of the following copy number states i.e. {homozygous deletion, heterozygous deletion, diploid and duplications}. In each state, the characteristics of the read counts and mate pair signals which are used for the mathematical modeling are described below:

#### Diploid

in this state, j^th^ sample carries two copies of the corresponding segment in the reference genome. Here, a mate pair which is generated from the j^th^ sample aligns to the reference genome with a normal insertion size, distributed with the clone library insertion size distribution. Also, number of reads which are mapped to the corresponding segment in the reference genome i.e. ${f}_{j}$ is assumed to have a Poisson distribution with parameter $\lambda$.

#### Heterozygous deletion

in this state, the j^th^ sample carries only one allele of the corresponding segment in the reference genome. Therefore, some mate pairs which are generated from the j^th^ sample align to the reference with a normal insertion size, distributed with the clone library insertion size distribution. Other mate pairs align to the reference genome much further apart than expected. In this state, read counts are also distributed with a Poisson distribution with a parameter of $\frac{\lambda }{2}$.

#### Homozygous deletion

in this state, both alleles are deleted from the j^th^ sample. Therefore, a high percentage of the mate pairs which are generated from this region will map to the reference genome much further apart than the expected insertion size distribution in the clone library. Also, we consider a Poisson distribution with a parameter of $\varepsilon \lambda$ for the read counts, where $\varepsilon$ is assumed to be a very small value.

#### Duplication

in this state, the sample genome carries more than two copies of the corresponding segment in the reference genome. However, mate pairs which are generated from duplicated regions will map to the reference with the insertion size distribution of the clone library. Read counts are also distributed with a Poisson distribution with a parameter of $i\,{\rm{\lambda }}/2$, for the samples carrying i copies.

### Mathematical modeling of mate pair insertion sizes and read counts

Consider a segment of the reference genome whose copy number in the j^th^ sample is of interest, j = 1, 2, …, k. Also, let ${n}_{j}$ denote the total number of mate pairs which are generated from the j^th^ sample and align to the reference genome with condition ii, as mentioned before. Also, ${n}_{j1}\,$denotes the number of mate pairs which are mapped to the reference with the insertion size distribution of the clone library and ${n}_{j2}$ denotes the number of mate pairs which are mapped to the reference much further apart, compared to the insertion sizes in the clone library. Clearly, ${n}_{j}={n}_{j1}+{n}_{j2}$. Here, we assumed that ${n}_{j1}$ is binomially distributed as follows:

1 $$p({n}_{j1},{n}_{j2})=(\begin{array}{c}{n}_{j1}+{n}_{j2}\\ {n}_{j1}\end{array}){\beta }_{i}^{{n}_{j1}}{(1-{\beta }_{i})}^{{n}_{j2}}$$

where, ${n}_{j1}=1,2,\ldots ,{n}_{j}$. In the above distribution, ${\beta }_{i}$ indicates the probability of observing a mate pair mapped to the reference with a clone library insertion size distribution, when sample genome is in the i^th^ CNV state. Where, i = 0, 1, 2, 3, …, m corresponds to {homozygous deletion, heterozygous deletion, diploid and duplications}. Also, the maximum copy number of a genomic segment is denoted by *m*, i.e. $i\le m$.

When j^th^ sample has i copies of the studied segment of the reference genome, read count ${f}_{j}$ follows a Poisson distribution with a parameter of ${\theta }_{i}\lambda$:

2 $$p(\,{f}_{j})={e}^{-{\theta }_{i}\lambda }\frac{{({\theta }_{i}\lambda )}^{{f}_{j}}}{{f}_{j}!}$$

where, ${{\rm{\theta }}}_{0}={\rm{\varepsilon }}{\rm{\lambda }}$, and ${{\rm{\theta }}}_{{\rm{i}}}=i{\rm{\lambda }}/2$, for ${\rm{i}}\ge 1$.

Also, from a total number of k samples, let ${{\rm{\alpha }}}_{{\rm{i}}}$ denote the percentage of samples which have i copies of the studied segment of the reference genome. Taking the above descriptions into account, the probability of observing $({f}_{j},{n}_{j1},{n}_{j2})$ in the j^th^ sample genome can be written as follows:

3 $$\begin{array}{rcl}{p}(\,{{f}}_{{\boldsymbol{j}}},{{n}}_{{\boldsymbol{j}}1},{{n}}_{{\boldsymbol{j}}2}) & = & \sum _{{\boldsymbol{i}}=0}^{{m}}{{\alpha }}_{{\boldsymbol{i}}}{p}({{f}}_{{j}},{{n}}_{{j}1},{{n}}_{{j}2}|{state}\,{i})\\  & = & \sum _{{\boldsymbol{i}}=0}^{{m}}{{\alpha }}_{{i}}{{e}}^{-{{\boldsymbol{\theta }}}_{{\boldsymbol{i}}}{\boldsymbol{\lambda }}}\frac{{({{\theta }}_{{\boldsymbol{i}}}{\lambda })}^{{{\boldsymbol{f}}}_{{\boldsymbol{j}}}}}{{{f}}_{{\boldsymbol{j}}}!}[(\begin{array}{c}{{n}}_{{\boldsymbol{j}}1}+{{n}}_{{\boldsymbol{j}}2}\\ {{n}}_{{\boldsymbol{j}}1}\end{array}){{\beta }}_{{\boldsymbol{i}}}^{{{\boldsymbol{n}}}_{{\boldsymbol{j}}1}}{(1-{{\beta }}_{{\boldsymbol{i}}})}^{{{n}}_{{\boldsymbol{j}}2}}]\end{array}$$

However, it should be added that in the above formulations ${n}_{j1}$ and ${n}_{j2}$ are not known and they depend on the unknown parameter ${\beta }_{i}$. Also, the estimation of ${n}_{j1}$ and ${n}_{j2}$, requires estimating the probability of each insertion size to be distributed with the clone library insertion size distribution. For this purpose, let ${o}_{jr}$ denote insertion size of the r^th^ mate pair which was generated from the j^th^ sample and was mapped to the studied segment of the reference genome. Where $r=1,\,2,\,\ldots ,\,\,{n}_{j}$ and j = 1, 2, …, k. Consequently, a random variable ${z}_{jr}$ is corresponded to each mate pair insertion size ${o}_{jr}$:

$${z}_{jr}=\{\begin{array}{l}1\,if\,{o}_{jr}\,comes\,from\,the\,insertion\,size\,distribution\,of\,the\,clone\,library\\ 0\,if\,{o}_{jr}\,comes\,from\,a\,shifted\,insertion\,size\,distribution\,of\,the\,clone\,library\end{array}$$

where, ${n}_{j1}=\sum _{r=1}^{{n}_{j}}{z}_{jr}$ and ${n}_{j2}=\sum _{r=1}^{{n}_{j}}(1-{z}_{jr})$. To estimate the expected value of ${n}_{j1}$ and ${n}_{j2}$, we calculate the probability of having a ${z}_{jr}$ equal to 1, for $r=1,\,2,\,\ldots ,\,\,{n}_{j}$ and $j=1,\,2,\,\ldots ,\,\,k$, see Supplementary file 1 for a detailed description.

Now, a Dirichlet prior distribution is defined for the parameter vector ${\boldsymbol{\alpha }}=({\alpha }_{0},\,{\alpha }_{1},\,\ldots ,\,{\alpha }_{m})$:

4 $${p}({\boldsymbol{\alpha }})\propto \prod _{{\boldsymbol{i}}=0}^{{\boldsymbol{m}}}{{\alpha }}_{{\boldsymbol{i}}}^{{{\boldsymbol{\gamma }}}_{{\boldsymbol{i}}}-1}$$

where, ${\alpha }_{0}=1-\sum _{i=1}^{m}{\alpha }_{i}\,$and ${\gamma }_{s}=\sum _{i=0}^{m}{\gamma }_{i}$. Also, the prior of each ${\beta }_{i}$ is considered to be a beta distribution as follows:

5 $${p}({{\beta }}_{{\boldsymbol{i}}})=\frac{{\Gamma }({{\nu }}_{{\boldsymbol{i}}1}+{{\nu }}_{{\boldsymbol{i}}2})}{{\Gamma }({{\nu }}_{{\boldsymbol{i}}1}){\Gamma }({{\nu }}_{{\boldsymbol{i}}2})}{{\beta }}_{{\boldsymbol{i}}}^{{{\boldsymbol{\nu }}}_{{\boldsymbol{i}}1}-1}{(1-{{\beta }}_{{\boldsymbol{i}}})}^{{{\boldsymbol{\nu }}}_{{\boldsymbol{i}}2}-1}$$

where, i = 0, 1, 2, …, m. Moreover, the prior distribution of $\lambda$ is considered to be a uniform distribution, over the interval of $(0,t)$, where t is large enough.

### Model parameters

There are a number of parameters in the above mathematical model which have to be estimated. These parameters include $\lambda$, the average read counts in a genomic segment of diploid state. The parameter vector ${\boldsymbol{\alpha }}=({\alpha }_{0},\,{\alpha }_{1},\,\ldots ,\,{\alpha }_{m})$ represents the percentage of samples with copy numbers 0, 1, …, m of the studied segment of the reference genome. Also, for a sample genome with copy number state *i*, ${\beta }_{i}$ indicates the proportion of the mate pairs which are mapped to the reference genome much further apart than expected under the clone library insertion size distribution.

The parameters of the prior distribution over ${\boldsymbol{\alpha }}=({\alpha }_{0},\,{\alpha }_{1},\,\ldots ,\,{\alpha }_{m})$ are given values based on information from genome-wide CNV percentage. Since a high percentage of genomic segments in each sample are expected to be in diploid state, ${\gamma }_{2}$ is given a value much higher than ${\gamma }_{i}$, $i\ne 2$. Also, a beta distribution is defined as a prior distribution over each ${\beta }_{i}$, i = 0, 1, 2, …, m. The parameters of the beta distribution i.e. ${\nu }_{i1}$ and ${\nu }_{i2}$ are given values based on the expected number of mate pairs which are mapped to the reference much further apart, compared to the clone library insertion size distribution. In genomic diploid state and segments with an elevated number of copies ${\nu }_{i1}\gg {\nu }_{i2}$, for i = 2, 3, …, m. In genomic segments with heterozygous deletion ${\nu }_{11}\cong {\nu }_{12}$ and in genomic segments with homozygous deletions ${\nu }_{01}\ll {\nu }_{02}$.

### Parameter estimation

MSeq-CNV applies the Expectation-Maximization (EM) algorithm, for parameter estimation. The parameter estimation details are given in Supplementary file 1.

### Parameter initialization in EM algorithm

$\lambda$ is initialized based on the number of reads that are expected to be generated from a genomic segment with diploid state, after taking the sequencing coverage into account. For example, for a coverage of 5×, a read length of 100 bp and genomic segments of length 100 bp, $\lambda$ is initialized with a value of 20. Also, ${\alpha }_{2}=0.90$, ${\alpha }_{i}=0.02$ for i = 0, 1, 3, 4, 5, and ${\beta }_{2}={\beta }_{3}={\beta }_{4}={\beta }_{5}=1$, ${\beta }_{0}=0$, and ${\beta }_{1}=0.5$ are taken as the start point of the parameters in the EM algorithm. In the j^th^ sample, ${\mu }_{j1},{\mu }_{j2}$ are initialized by comparing the mate pair insertion sizes with the clone library insertion size distribution.

In this study, we have considered a segment size of 150 bps in the simulated data analysis and a segment size of 100 bps in the real data analysis. Considering a longer segment size decreases the running time of the algorithm, with the cost of lower resolution. The methods which are compared to MSeq-CNV are also implemented with the same segment size, as MSeq-CNV. However, there are other methods which are specialized in detecting genomic rearrangements and tandem duplications using paired reads which get the nucleotide resolution breakpoint^25,29^.

### Data availability

BAM (Binary Alignment/Map) files of the alignment of the mate pair reads to the build 36 (hg18) of the human reference genome are available at ftp://ftp-trace.ncbi.nih.gov/1000genomes/ftp/. R program of MSeq-CNV and a detailed procedure for its running is available at https://github.com/CNVdetection/MSeq-CNV. The list of detected CNVs in the studied individuals is also submitted to this webpage.

## Results

### Implementation To Real Data of The Human Reference Chromosome

We have constructed 40 sample genomes with implanted CNVs, from chromosome 3 of the human reference genome. After duplicating chromosome 3 of the reference genome, it was altered with implanted CNVs of length 250 bp, 500 bp, 750 bp, 1 kb, 1.5 kb, 2 kb, 2.5 kb, 3 kb, 3.5 kb, 4 kb, 4.5 kb, 5 kb. The position of each CNV is randomly chosen so that CNVs do not overlap along chromosome. After determining the CNV positions on the reference genome, CNVs are implanted into each sample genome.

Indeed, for each CNV region which is implanted in the reference genome, distributions and characteristics of CNVs across sample genomes are determined based on a previous analysis of the HapMap individuals.

Based on the characteristics of the HapMap individuals, 80% of the implanted CNVs were of type loss in which deletions occurs in some sample genomes. Also, 15% of the CNV regions were of type gain in which some sample genomes have an elevated number of copies. The other 5% of the implanted CNV regions were of type mixed in which sample genomes may either have copy loss or copy gain.

In each CNV region, the copy number of each sample was also drawn from the copy number distribution in HapMap individuals. For a genomic loss region, a sample has copy numbers 2, 1, and 0 with probabilities 0.8, 0.15 and 0.05, respectively. For a genomic gain region, a sample has copy numbers 2, 3, 4, and 5 with probabilities 0.85, 0.08, 0.06 and 0.01, respectively. Also, for a CNV region of type mixed, a sample has copy numbers 0, 1, 2, 3, 4 with probabilities 0.04, 0.16, 0.67, 0.11 and 0.02, respectively.

After constructing the sample genomes, MAQ is applied for generating mate pair reads from each sample genome. Mate pairs are then mapped to the human reference genome. After dividing the human reference genome into segments of length 150 bp, MSeq-CNV is applied for detecting CNVs in the corresponding segments of the constructed sample genomes. In Table 1, the performance of MSeq-CNV is reported for each CNV state i.e. homozygous deletion, heterozygous deletion, and duplications, for a genome-wide sequencing coverage of 10×.

**Table 1:** Precision and recall values of MSeq-CNV are evaluated in 1,120,000 genomic segments of length 150 bp (168 million base pairs are evaluated), for a genome-wide coverage of 10×. In columns 3 to 8, predicted states are reported versus the real states of the genomic segments.

		Real state								
		Heterozygous deletion	Homozygous deletion	Diploid	3 copies	4 copies	5 copies	Sum	Precision	Recall
Predicted state	Heterozygous deletion	26,807	545	4,529	26	12	2	31,921	0.84	0.94
	Homozygous deletion	1,682	86,979	20,474	172	74	24	109,405	0.80	0.99
	Diploid	0	215	954,226	3,106	549	73	958,169	1.00	0.97
	Duplication, 3 copies	0	344	3,124	8,953	554	45	13,020	0.69	0.71
	Duplication, 4 copies	0	0	0	371	5,460	96	5,927	0.92	0.75
	Duplication, 5 copies	0	0	0	0	654	904	1,558	0.58	0.79
	Sum	28,489	88,083	982,353	12,628	7,303	1,144			

The performance of MSeq-CNV is also compared to the central CNV detection tools i.e. rSW-seq, CNV-seq and cnMOPS. These tools are selected for comparisons because of their high resolution and their capability in detecting both genome-wide deletions and duplications^37^. It should be added that rSW-seq and CNV-seq are not capable of detecting the digitized copy number of genomic regions i.e. these tools do not discriminate heterozygous deletions from homozygous deletions. However, MSeq-CNV resembles cn.MOPS in detecting the digitized copy number of each CNV region.

For calculating precisions and recalls, the whole simulation study was repeated five times for each setting and the average results across five repeats are summarized in Table 2. In this table, for a genome-wide sequencing coverage of 1×, 5×, 10×, MSeq-CNV is compared to the other tools in each genomic state i.e. homozygous deletion, heterozygous deletion, diploid and duplications. The F-score which is the harmonic mean of the precision and recall values are also calculated in Table 2, for each CNV state.

**Table 2:** MSeq-CNV in comparison with rSW-seq, CNV-seq and cn.MOPS, for whole-genome sequencing coverage of 1×, 5×, 10×. The average precision and recall values are calculated across five different repeats of the whole study. The highest F-scores are indicated in bold.

		Coverage								
		1×			5×			10×		
		precision	recall	F-score	precision	recall	F-score	precision	recall	F-score
Duplication	MSeq-CNV^*^	0.31	0.36	**0.33**	0.69	0.80	**0.74**	0.79	0.82	**0.80**
	MSeq-CNV (3 copies)	0.01	0.01	0.01	0.49	0.57	0.53	0.63	0.72	0.67
	MSeq-CNV (4 copies)	0.26	0.37	0.31	0.69	0.59	0.64	0.82	0.66	0.73
	MSeq-CNV (5 copies)	0.25	0.55	0.34	0.32	0.80	0.46	0.39	0.81	0.53
	rSW-seq	0.00	0.00	0.00	0.76	0.43	0.55	0.87	0.72	0.79
	CNV-seq	0.12	0.43	0.19	0.72	0.58	0.64	0.97	0.60	0.74
	cn.MOPS	0.00	0.00	0.00	0.00	0.00	0.00	0.00	0.00	0.00
Deletion	MSeq-CNV (heterozygous deletion)	0.84	0.96	0.90	0.85	0.93	0.89	0.94	0.94	0.94
	MSeq-CNV (homozygous deletion)	0.80	0.85	0.82	0.81	0.98	0.89	0.86	0.99	0.92
	MSeq-CNV (hetero + homo)**	0.83	0.90	**0.86**	0.83	0.99	**0.90**	0.89	1.00	**0.94**
	rSW-seq	0.00	0.00	0.00	0.87	0.56	0.68	0.92	0.87	0.89
	CNV-seq	0.66	0.58	0.62	0.98	0.78	0.87	0.99	0.87	0.93
	cn.MOPS	1.00	0.23	0.37	1.00	0.23	0.37	1.00	0.23	0.37
Diploid	MSeq-CNV	0.98	0.96	**0.97**	0.99	0.97	**0.98**	1.00	0.98	**0.99**
	rSW-seq	0.87	1.00	0.93	0.93	0.98	0.95	0.97	0.99	0.98
	CNV-seq	0.94	0.90	0.92	0.97	0.99	**0.98**	0.98	1.00	**0.99**
	cn.MOPS	0.90	1.00	0.95	0.90	1.00	0.95	0.90	1.00	0.95

^*^Precision, recall and F-score are evaluated over all genomic segments with amplifications i.e. 3, 4 and 5 copies.

**Hetero + homo stands for copy losses, both heterozygous and homozygous deletions.

As shown in Table 2, for all coverage values and according to the F-score, the performance of MSeq-CNV has been superior to the compared tools in detecting genomic regions with deletions and duplications. In regions with diploid copies, MSeq-CNV outperformed the compared tools for a coverage of 1×. Also for a coverage of 5× and 10×, MSeq-CNV and CNV-seq are both ranked as the best tools in detecting regions with diploid copies.

In Table 3, the overall performance of MSeq-CNV is compared to the other tools. To calculate the overall performance of each tool in estimating the correct copy number state, number of nucleotides whose states were correctly predicted is divided by the genome length. As indicated in Table 3, the overall performance of the MSeq-CNV is superior to rSW-seq, CNV-seq and cnMOPS, for a coverage of 1× and 5×. Also, MSeq-CNV and CNV-seq outperformed rSW-seq and cn.MOPS with an overall accuracy of 0.98, for a 10× coverage.

**Table 3:** The overall performance of the MSeq-CNV in comparison with rSW-seq, CNV-seq and cn.MOPS. For each method, the average of overall accuracies over five different runs of the whole study is given in each cell. For each coverage, the highest accuracies are indicated in bold.

		Coverage		
		1×	5×	10×
All regions**	MSeq-CNV	**0.94**	**0.96**	**0.98**
	rSW-seq	0.87	0.92	0.96
	CNV-seq	0.86	0.95	**0.98**
	cn.MOPS	0.90	0.90	0.90

**Number of nucleotides whose states were correctly predicted is divided by the total number of genomic nucleotides (genome length).

Performance of MSeq-CNV is also evaluated in terms of allele frequency of CNVs. As shown in Table 4, in CNV regions with copy loss, accuracy does not change with an increase in allele frequency i.e. MSeq-CNV is accurate in detecting genomic deletions. However, in CNV regions of type copy gain or mixed, overall accuracies decrease with an increase in allele frequency. This is associated with lower accuracies in detecting genomic duplications, compared to the other genomic regions.

**Table 4:** The overall accuracy of MSeq-CNV, in terms of allele frequency in genomic regions with CNVs. Allele frequencies are categorized into 4 groups i.e. 0–5, 6–10, 11–15, and 16–25, in the simulated data of 40 samples.

		Allele Frequency			
		0–5	6–10	11–15	16–25
CNV region	Copy Loss	0.99	0.99	0.99	0.99
	Copy Gain	0.97	0.93	0.86	0.86
	Mix (Copy gain + Copy loss)	0.96	0.96	0.96	0.94

**Figure 1:**
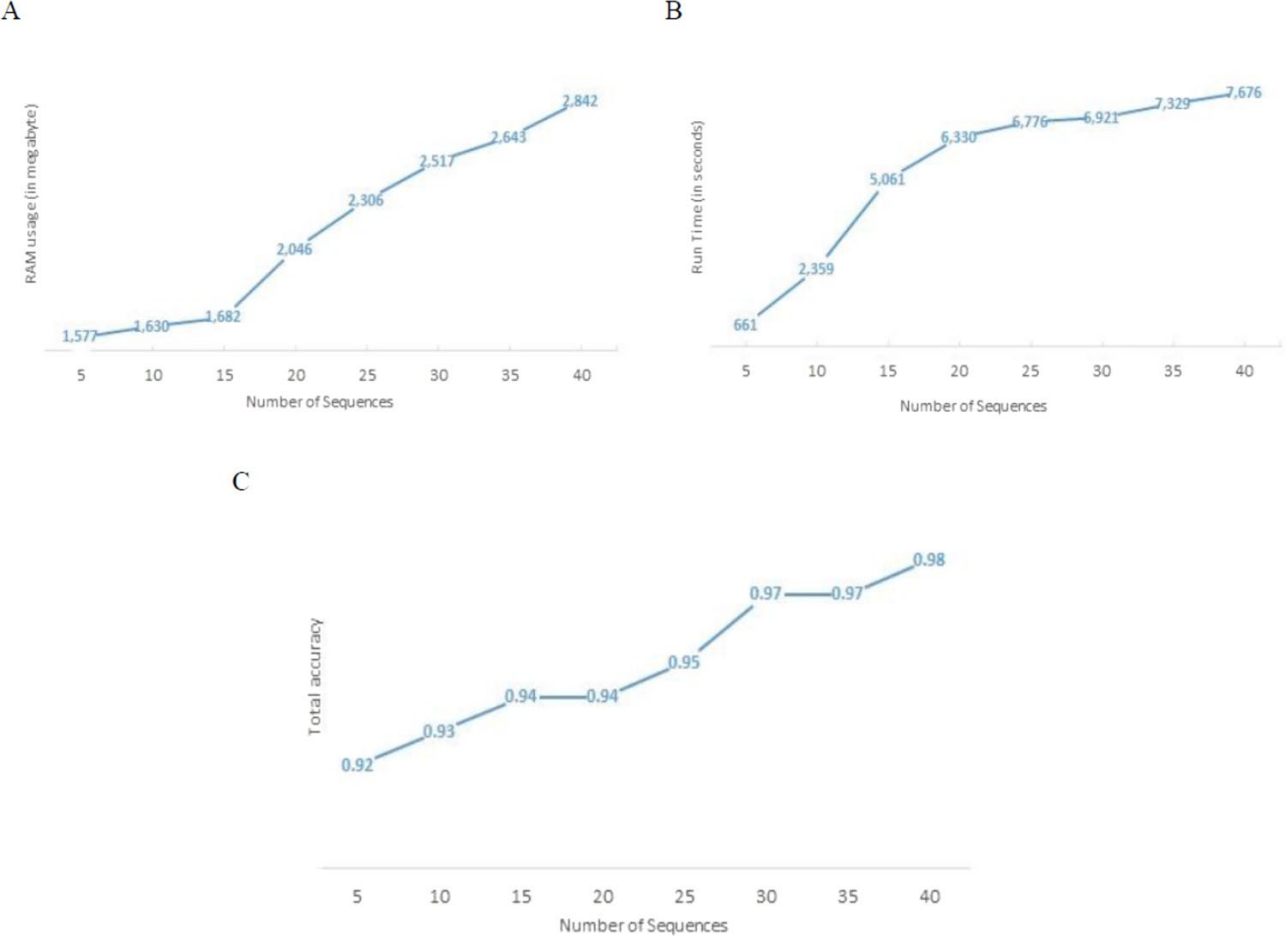
Ram usage, run time and accuracy of MSeq-CNV is reported, in terms of sequence numbers, in a 5 Mega bp of the genomic region and for a coverage of 10×. These results are achieved using parallel programming version of the MSeq-CNV on a 64-bit windows operating system with Intel Core(TM) i7-4710HQ CPU @3.5 GHz processor. (**A**) Ram usage of MSeq-CNV in terms of sequence numbers. **(B**) Run time of MSeq-CNV in terms of sequence numbers. (**C**) The overall accuracy of MSeq-CNV, in terms of number of analyzed individuals.

Figure 1 shows the RAM usage, running time and overall accuracy of MSeq-CNV in terms of sequence numbers, i.e. number of individuals which are compared to each other, for 10× sequencing coverage. To obtain these results, 4 computer cores were applied for running the parallel programming version of the MSeq-CNV, on a 64-bit windows operating system with Intel Core(TM) i7-4710HQ CPU @3.5 GHz processor. As shown in Fig. 1A,B, RAM usage and running time of MSeq-CNV both increase, with an increase in sequence numbers. However, as shown in Fig. 1C, analyzing more sample genomes at a time has a positive effect on the overall accuracy of MSeq-CNV.

### Results From The High-Coverage Data of The 1000 Genomes Project

MSeq-CNV is applied for the CNV detection in the genome of six HapMap individuals. These genomes which are sequenced with a high coverage as part of the 1000 Genomes Project (http://www.1000genomes.org) consist of a CEU trio of European ancestry (NA12891, NA12892 and NA12878) and a YRI trio of Yoruba Nigerian ethnicity (NA19238, NA19239 and NA19240).

BAM (Binary Alignment/Map) files of the alignment of the mate pair reads to the build 36 (hg18) of the human reference genome are downloaded from ftp://ftp-trace.ncbi.nih.gov/1000genomes/ftp/. Mate pair reads with low mapping qualities (Q < 25) are then filtered out using SAMtools (samtools.sourceforge.net). Then, MSeq-CNV is applied for detecting deletions and duplications in the genome of CEU and YRI individuals, simultaneously.

In past studies^44^, to reduce the false discovery rate, CNVs’ callset were heavily pre-filtered and only a high confident set of CNVs were reported. In our Bayesian framework, to reduce the false positive rate, we report a set of CNVs with high posterior probabilities. Indeed, those CNVs with posterior probabilities lower than a fixed threshold i.e. 0.5 are filtered out from the callset.

In Table 5, number of CNVs and their total size (in Mega bp) are reported for deletion and duplication calls with at least 1 kb in size. As indicated in Table 5, numbers of CNVs in CEU trio NA12891, NA12892 and NA12878 are respectively 249, 404 (421.284 Mega bp), 248, 889 (390.479 Mega bp) and 249, 162 (410.194 Mega bp). Also, numbers of CNVs in YRI trio NA19238, NA19239 and NA19240 are respectively 278, 027 (459.610 Mega bp), 246, 229 (453.394 Mega bp) and 251, 269 (488.895 Mega bp). Also, similar to previous estimations^45^, CNV calls in NA12891, NA12892, NA12878, NA19238, NA19239 and NA19240 respectively cover 13.02%, 12.07%, 12.68%, 14.21%, 14.02% and 15.11% of the human genome.

**Table 5:** Number of CNV calls which are made by MSeq-CNV in the six high-coverage sequencing data the 1000Genomes Project i.e. NA12891, NA12892, NA12878 (CEU trio), NA19238, NA19239, NA19240 (YRI trio) and in the low-coverage sequencing data of NA12761, NA12762, and six individuals from the Simons Genome Diversity Project (SGDP).

	Individual	Number of deletion calls	Total size of deletion calls in Mega bp	Number of duplication calls	Total size of duplication calls in Mega bp	Number of CNVs	Total size of CNV calls in Mega bp
CEU trio, 1000Genomes Project, high-coverage sequencing	NA12891	79,209	143.442	170,195	277.842	249,404	421.284
	NA12892	85,496	151.703	163,393	238.777	248,889	390.479
	NA12878	86,628	155.460	162,534	254.734	249,162	410.194
Average per individual		83,778	150.201	165,374	257.117	249,152	407.319
YRI trio, 1000Genomes Project, high-coverage sequencing	NA19238	116,971	209.685	161,056	249.925	278,027	459.610
	NA19239	81,688	156.520	164,541	296.874	246,229	453.394
	NA19240	76,169	146.339	175,100	342.556	251,269	488.895
Average per individual		91,609	170.848	166,899	296.452	258,508	467.300
1000Genomes Project, low-coverage sequencing	NA12761	56,415	88.675	89,047	102.820	145,462	191.495
	NA12762	47,541	76.609	71,533	92.730	119,074	169.339
Average per individual		51,978	82.642	80,290	97.775	132,268	180.417
American, SGDP	USA, LP6005592-DNA_H03	38,172	65.276	133,238	237.788	171,410	303.064
Asian, SGDP	TAIWAN, LP6005442-DNA_E07	70,114	102.897	127,321	220.741	197,435	323.638
	TAIWAN, LP6005443-DNA_G05	52,834	83.333	130,605	234.064	183,439	317.397
	INDIA, LP6005519-DNA_A04	131,537	186.819	130,868	244.590	262,405	431.409
	INDIA, LP6005519-DNA_A05	204,704	293.091	137,205	252.344	341,909	545.435
Average per individual		114,797	166.535	131,500	237.935	246,297	404.469
European, SGDP	FINLAND, LP6005592-DNA_D01	56,491	88.131	129,346	235.698	185,837	323.829

The average number of CNVs in YRI trio i.e. 258, 508 (467.300 Mega bp) is slightly higher than the average calls in CEU i.e. 249, 152 (407.319 Mega bp). Also, as indicated in Table 5, the average number of deletion and duplication calls in YRI individuals (91, 609 and 166, 899) are both more than CEU trio (83, 778 and 165, 374), indicating the increased diversity of the African individuals in comparison with CEUs^46^. Moreover, in the studied individuals genomic deletions are less common compared to duplications^44,46,47^.

**Figure 2:**
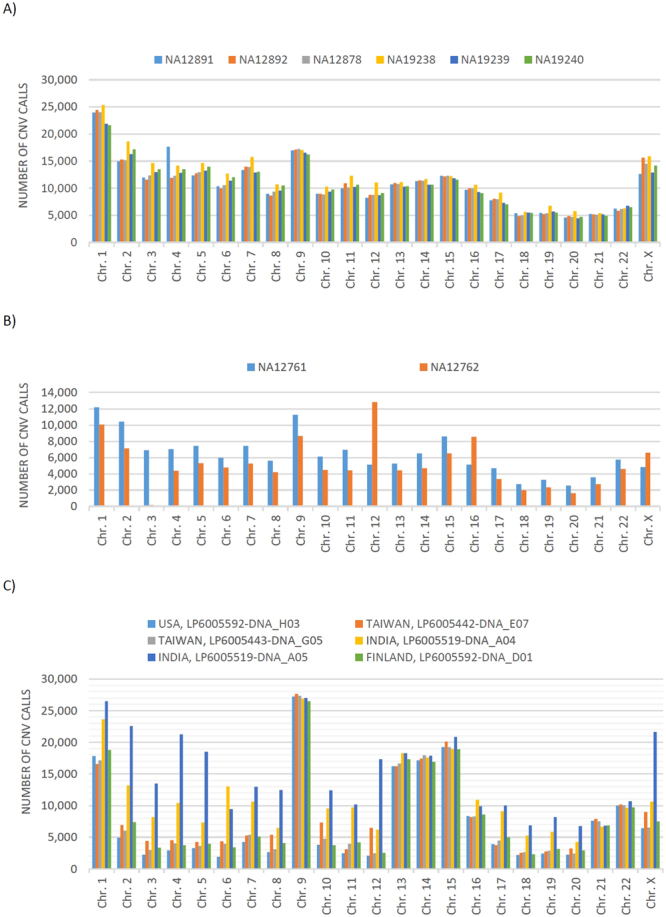
Number of CNV calls which are made by MSeq-CNV. (**A**) In the high-coverage sequencing data of HapMap individuals NA12891, NA12892, NA12878, NA19238, NA19239 and NA19240, from 1000 Genomes Project. (**B**) In the low-coverage sequencing data of NA12761 and NA12762, from 1000Genomes Project. (**C**) In the six individuals from the Simons Genome Diversity Project (SGDP).

Total number of CNV calls in each chromosome is plotted in Fig. 2A, for each HapMap individual. Numbers of deletion and duplication calls are also given in Table S.1, for each chromosome. See Table S.2 for the size (in Mega bp) of deletion and duplication calls.

To investigate the validity of the CNV calls, their overlap with the Database of Genomic Variants (DGV)^48^, http://dgv.tcag.ca/dgv/ is studied. DGV includes 8, 599 CNVs from 40 HapMap individuals which are validated experimentally using aCGH methods.

The overlap of the detected CNVs with DGV are determined by the number of calls and also by the size of overlap, in base pairs. CNV calls in CEU trio NA12891, NA12892 and NA12878 overlap with DGV respectively with a ratio of 0.61, 0.62 and 0.61, for the number of calls. Also, a base which is called as a CNV in NA12891, NA12892 and NA12878 overlap with a base in DGV respectively with a ratio of 0.60, 0.61 and 0.61. The YRI individuals NA19238, NA19239 and NA19240 overlap with DGV respectively with a ratio of 0.62, 0.61 and 0.62 for the number of calls, and 0.61, 0.59 and 0.60 for the base pairs. Therefore, more than a half of CNV calls which are made by MSeq-CNV are previously validated using aCGH methods.

**Figure 3:**
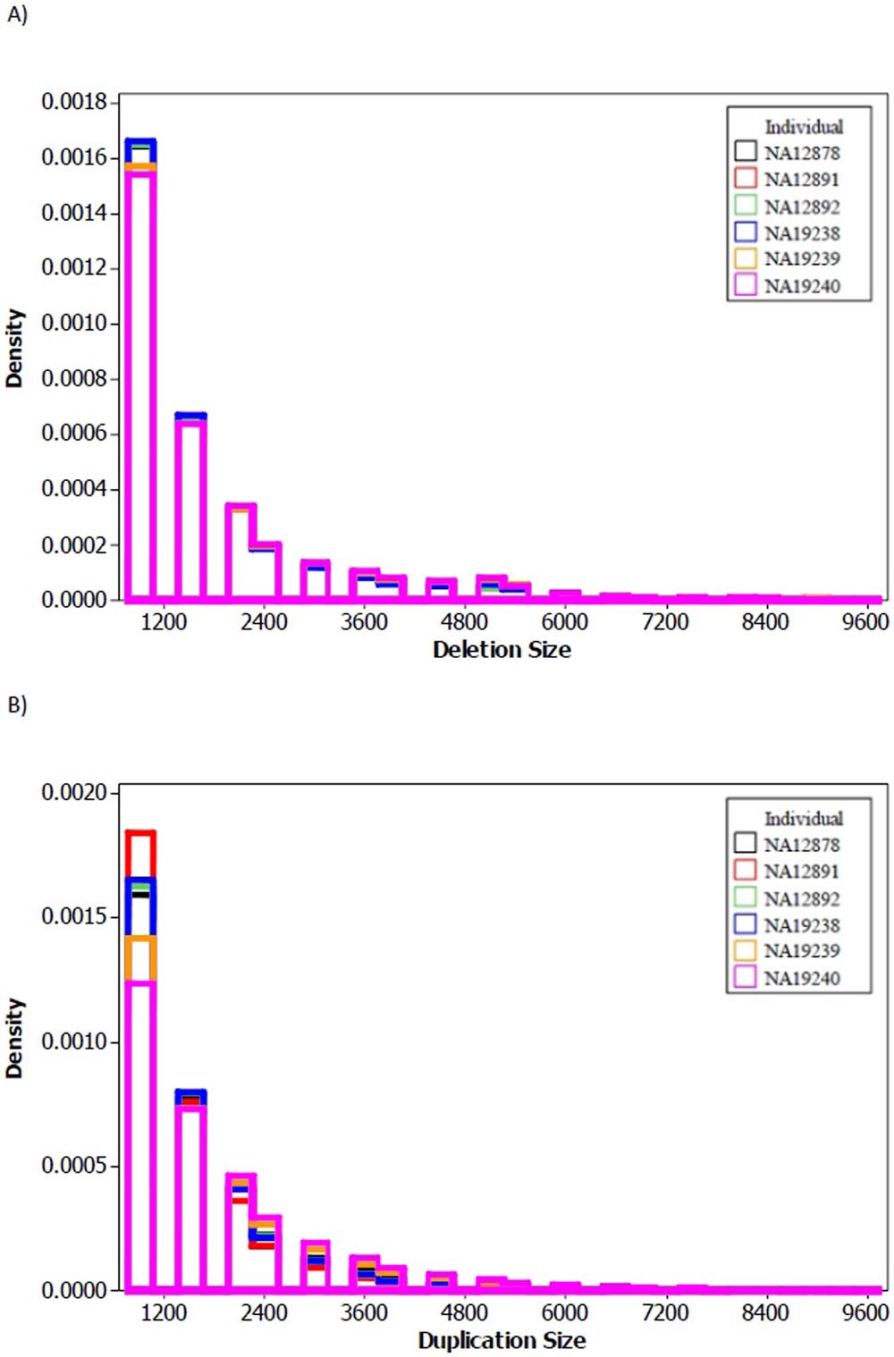
The size distribution of the deletion and duplication calls – made by MSeq-CNV - in six individuals with high-coverage sequencing from 1000Genomes Project. (**A**) Deletion size distributions, (**B**) Duplication size distributions.

Size distribution of CNVs are also shown in Fig. 3A,B, respectively for the deletion and duplication calls. Clearly, the numbers of deletion and duplication calls decrease exponentially, with an increase in CNV size. As shown in Fig. 3A, deletion size distributions almost overlap in all studied individuals. Duplication size distributions are also very similar in all individuals, with CEU individuals having more CNVs of smaller sizes.

**Figure 4:**
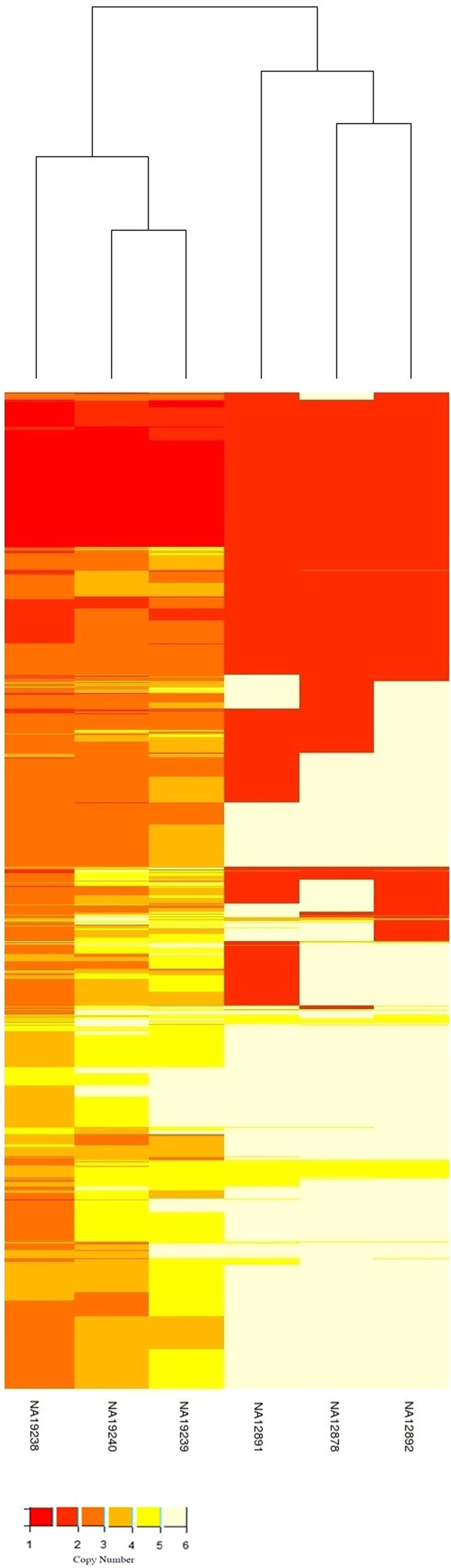
The hierarchical clustering algorithm is applied to the matrix of CNV regions which are detected using MSeq-CNV in the genome of HapMap individuals NA12891, NA12892, NA12878, NA19238, NA19239 and NA19240. Based on the CNV information, the hierarchical clustering algorithm has correctly segregated the ancestry of the six individuals in two groups i.e. a group includes CEU trio and a group includes YRI trio.

Moreover, we applied the hierarchical clustering algorithm to the matrix of CNV regions which are identified in the genome of six HapMap individuals. As shown in Fig. 4, although no information about the individual’s identities are used in the hierarchical clustering, the algorithm has correctly segregated the ancestry of the six individuals in two groups. While one group includes the CEU individuals NA12891, NA12892 and NA12878 with European ancestry, the other group includes YRI individuals NA19238, NA19239 and NA19240 with Nigerian ancestry.

### Results From The Low-Coverage Data of The 1000 Genomes Project

MSeq-CNV is also applied for the CNV detection form the low-coverage data of two individuals i.e. NA12761 and NA12762, from 1000 Genome project. After downloading BAM files of the alignment of mate pair reads to the human reference genome, mate pairs with low mapping qualities (Q < 25) are filtered out.

MSeq-CNV called a total number of 145, 462 (191.495 Mega bp) and 119, 074 (169.339 Mega bp) CNVs in the genomes of NA12761 and NA12762, respectively. Also, in both individuals, genomic deletions are less common compared to the duplications^44,46,47^. Details of deletion and duplication calls are given in Table 5 and Table S.3.

The low number of CNV calls in the genome of NA12761 and NA12762 is potentially associated with lower accuracies in detecting genomic CNVs, especially duplications, from low-coverage sequencing data of NA12761 and NA12762 (see Table 2).

The overall number of detected CNVs in each chromosome is shown in Fig. 2B, for NA12761 and NA12762. Detected CNVs in NA12761 and NA12762 overlap with DGV respectively with a ratio of 0.65 and 0.66 for the number of calls, and 0.66 and 0.67 for base pairs.

### Results From The Simons Genome Diversity Project (SGDP)

MSeq-CNV is also applied for CNV detection in the genome of six individuals from the Simons Genome Diversity Project^49^ i.e. LP6005592-DNA_H03 (USA), LP6005442-DNA_E07 (Taiwan), LP6005443-DNA_G05 (Taiwan), LP6005519-DNA_A04 (India), LP6005519-DNA_A05 (India), and LP6005592-DNA_D01 (Finland).

As indicated in Table 5, in the analyzed individuals from SGDP, the lowest number of CNVs are called in the LP6005592-DNA_H03 (USA)^46^ (171, 410 CNVs with a total size of 303.064 Mega bp). Two East Asian individuals LP6005443-DNA_G05, LP6005442-DNA_E07(form TAIWAN) and West Eurasian individual LP6005592-DNA_D01 (from FINLAND) are the next, respectively with a total number of 183, 439 (317.397 Mega bp), 197, 435 (323.638 Mega bp), 185, 837 (323.829 Mega bp) calls.

The highest number of CNVs are detected in the South Asian individuals LP6005519-DNA_A04 and LP6005519-DNA_A05 (from INDIA) respectively with a total number of 262, 405 (431.409 Mega bp) and 341, 909 (545.435 Mega bp) calls. Extensive CNVs in Indian individuals, which is as many as YRI trio, were also previously reported in the admixed Indian population of African ancestry^47,50^, to adopt with environmental conditions.

Details of deletion and duplication calls are given in Table S.4 and Table S.5. The overall number of detected CNVs in each chromosome is shown in Fig. 2C, for each individual.

Detected CNVs in LP6005592-DNA_H03, LP6005442-DNA_E07, LP6005443-DNA_G05 LP6005519-DNA_A04 and LP6005519-DNA_A05, and LP6005592-DNA_D01 overlap with DGV respectively with a ratio of 0.61, 0.62, 0.61, 0.60, 0.62, and 0.62, for the number of calls and 0.60, 0.60, 0.59, 0.60, 0.59, and 0.61, for the base pairs.

### Applications and Limitations

The MSeq-CNV can be applied for detecting the recurrent genome-wide CNVs from NGS data in the diploid genome of human and other organisms, as well. However, the current version of MSeq-CNV is not capable of detecting CNVs in the sequencing data of a haploid genome. The input NGS data for the MSeq-CNV are possibly the mate pair reads which are collected from sequencing with multiple platforms, multiple individuals and experimental conditions.

Although the current version of the MSeq-CNV is limited to the whole genome shotgun sequencing, further work is in progress to adopt MSeq-CNV with the exome or gene panel sequencing data.

Also, as mentioned above, the other attractive feature of the MSeq-CNV is in constructing the ancestry of the sequenced individuals, based on the detected CNV matrix.

## Discussion

In this article we proposed MSeq-CNV as a new tool for detecting genome-wide deletions and duplications from sequencing of multiple samples. Simultaneous analysis of multiple samples allows detecting common CNVs which are shared by complex diseases. Also, read count variations which occur due to the sequencing noise can be detected by the analysis of several samples together. MSeq-CNV applies a novel probabilistic framework for modeling the read depth and insertion size signals, together.

The overall performance of MSeq-CNV has been superior to the central CNV detection tools such as rSW-seq, CNV-seq and cnMOPS. Specially, for a coverage of 1× which is fairly low, the overall performance MSeq-CNV has been considerably higher than the compared tools. Reaching a high performance in low coverage data is an advantage of MSeq-CNV. In future, CNV detection tools which rely on a low-coverage sequencing are more relevant^37,38^. Indeed, a low coverage sequencing is common in many individuals e.g. in the 1000 Genomes Project the shotgun sequencing of 179 individuals is carried out with a coverage of 2× to 4×^41^.

The MSeq-CNV works with the empirical distribution of the insertion sizes in clone library. Therefore, MSeq-CNV is robust to deviations from the theoretical insertion size distribution which occurs due to several artifacts, attributed to the library-preparation protocols.

## Electronic supplementary material

Supplementary file 1
