# Supplementary material for: MSeq-CNV: accurate detection of Copy Number Variation from Sequencing of Multiple samples
Source: Sci Rep. 2018 Mar 5;8:4009. doi: 10.1038/s41598-018-22323-8 (PMC5838159; doi:10.1038/s41598-018-22323-8)
Supplement: Supplementary file 1 [file 41598_2018_22323_MOESM1_ESM.pdf]

MSeq-CNV: accurate detection of Copy Number Variation from Sequencing of Multiple samples

Supplementary file 1

Seyed Amir Malekpour [1], Hamid Pezeshk[1,2,3] * and Mehdi Sadeghi[4]
[1]School of Mathematics, Statistics and Computer Science, College of Science, University of Tehran, Tehran, Iran, [2]School of Biological Sciences, Institute for Research in Fundamental Sciences, Tehran, Iran, [3]Current address: Department of Mathematics and Statistics, Concordia University, Montreal, Canada, [4]National Institute of Genetic Engineering and Biotechnology, Tehran, Iran.

*To whom correspondence should be addressed.
Contact: a.malekpour@ut.ac.ir, pezeshk@khayam.ut.ac.ir, pezeshk@ut.ac.ir, sadeghi@nigeb.ac.ir

Supplementary information on parameter estimation

Since there are no data for training the model parameters, we apply the unsupervised learning algorithm of Expectation-Maximization (EM), for this purpose. First, let D_j denote the observation vector in the jth sample genome i.e. (f_j, n_{j1}, n_{j2}). Also, let $q = (q_1, q_2, \dots, q_k)$ denote the true copy number in a segment of the sample genomes, corresponding to the studied segment in reference genome. Now, the Q function can be written as follows:

$$
\begin{aligned}
Q(\theta,\theta^g) &= \sum_q \sum_z \sum_{j=1}^{k} \left[\log p(\boldsymbol{\alpha},\boldsymbol{\beta},\lambda \mid D_j,\mathbf{z},q)\right] p(D_j,\mathbf{z},q \mid \theta^g) \\
&= \sum_q \sum_z \sum_{j=1}^{k} \left[\log p(D_j,\mathbf{z},q \mid \boldsymbol{\alpha},\boldsymbol{\beta},\lambda) p(\boldsymbol{\alpha}) p(\boldsymbol{\beta})\right] p(D_j,\mathbf{z},q \mid \theta^g) \\
&= \sum_i \sum_z \sum_{j=1}^{k} log \left[\alpha_i e^{-\theta_i \lambda} \frac{(\theta_i \lambda)^{f_j}}{f_j!} \left[\binom{n_j}{n_{j1}} \beta_i^{\sum_{r=1}^{n_j} z_{jr}} (1 \right.\right. \\
&\quad \left.\left. - \beta_i)^{\sum_{r=1}^{n_j} (1 - z_{jr})} \right]\right] p(D_j,\mathbf{z},q = i \mid \theta^g) + \sum_q \sum_z \sum_{j=1}^{k} \log p(\boldsymbol{\alpha})\, p(D_j,\mathbf{z},q \mid \theta^g) \\
&\quad + \sum_q \sum_z \sum_{j=1}^{k} \log p(\boldsymbol{\beta})\, p(D_j,\mathbf{z},q \mid \theta^g) = \\
&= \sum_i \sum_z \sum_{j=1}^{k} \left[log \alpha_i - \theta_i \lambda + f_j \log \theta_i \lambda - \log f_j! + \log \binom{n_j}{n_{j1}} + \sum_{r=1}^{n_j} z_{jr} \log \beta_i \right. \\
&\quad \left. + \sum_{r=1}^{n_j} (1 - z_{jr}) \log(1 - \beta_i) \right] p(D_j,\mathbf{z},q = i \mid \theta^g) + \sum_{j=1}^{k} \left[\sum_{i=0}^{m} (\gamma_i - 1) \log \alpha_i \right] p(D_j \mid \theta^g) \\
&\quad + \sum_{j=1}^{k} \left[\sum_{i=0}^{m} [(\nu_{i1} - 1) \log \beta_i + (\nu_{i2} - 1) \log(1 - \beta_i)] \right] p(D_j \mid \theta^g)
\end{aligned}
$$

In above equation, $n_j = n_{j1} + n_{j2}$ and θ^g is a vector which represents the estimated values of all model parameters, in the gth iteration of the EM algorithm. Also, the true parameter values are denoted by θ. Now, we have to maximize the above Q function, with respect to each parameter. After taking the derivative of the Q function with respect to λ:

$\frac{\partial Q(\theta,\theta^g)}{\partial \lambda} = \sum_i \sum_z \sum_{j=1}^{k} \left[-\theta_i + \frac{f_j}{\lambda} \right] p(n_j, \mathbf{z}, q = i | \theta^g) = 0$

Then, we reach the following equation to re-estimate λ in the gth iteration of the EM algorithm:

$\lambda^{g+1} = \frac{\sum_{j=1}^{k} f_j p(D_j|\theta^g)}{\sum_{j=1}^{k} \sum_i \theta_i p(D_j, q = i|\theta^g)} = \frac{\sum_{j=1}^{k} f_j p(D_j|\theta^g)}{\sum_{j=1}^{k} \sum_i \theta_i p(q = i|D_j, \theta^g) p(D_j|\theta^g)}$

To estimate α_i, the restriction of the $\sum_{i=0}^{m} \alpha_i = 1$ is introduced into the Q function, using the Lagrange multiplier ψ. Then, after taking the derivative of the Q with respect to α_i:

$\frac{\partial Q(\theta,\theta^g)}{\partial \alpha_i} = \sum_{j=1}^{k} \frac{1}{\alpha_i} p(D_j, q = i|\theta^g) + \sum_{j=1}^{k} \frac{1}{\alpha_i} (\gamma_i - 1)\, p(D_j|\theta^g) + \psi = 0$

This gives the following formula for re-estimating α_i in the gth iteration of the EM algorithm:

$\alpha_i^{g+1} = \frac{\sum_{j=1}^{k} p(D_j, q = i|\theta^g) + (\gamma_i - 1) \sum_{j=1}^{k} p(D_j|\theta^g)}{\sum_{j=1}^{k} p(D_j|\theta^g)(1 + (\gamma_s - m))}$

Also, by taking the derivative of the Q function with respect to β_i we reach:

$$\frac{\partial Q(\theta,\theta^g)}{\partial \beta_i} = \sum_z \sum_{j=1}^{k} \left[\sum_{r=1}^{n_j} \frac{z_{jr}}{\beta_i} + \sum_{r=1}^{n_j} \frac{-(1 - z_{jr})}{1 - \beta_i} \right] p(D_j, \mathbf{z}, q = i|\theta^g) + \sum_{j=1}^{k} \left[\frac{(\nu_{i1} - 1)}{\beta_i} - \frac{(\nu_{i2} - 1)}{1 - \beta_i} \right] p(D_j|\theta^g) = 0$$

This results in having:

$\beta_i^{g+1} = \frac{\sum_z \sum_{j=1}^{k} \sum_{r=1}^{n_j} z_{jr} p(D_j, \mathbf{z}, q = i|\theta^g) + (\nu_{i1} - 1) \sum_{j=1}^{k} p(D_j|\theta^g)}{\sum_{j=1}^{k} n_j p(D_j, q = i|\theta^g) + (\nu_{i1} + \nu_{i2} - 2) \sum_{j=1}^{k} p(D_j|\theta^g)}$

To compute the value of $p(D_j, \mathbf{z}, q = i|\theta^g)$ in the above equation, we have:

$$\begin{aligned}
\sum_z \sum_{j=1}^{k} \sum_{r=1}^{n_j} z_{jr} p(D_j, \mathbf{z}, q = i|\theta^g) &= \sum_z \sum_{j=1}^{k} \sum_{r=1}^{n_j} z_{jr} p(D_j, \mathbf{z}, q = i|\theta^g) \\
&= \sum_{j=1}^{k} \sum_{r=1}^{n_j} p(D_j, z_{jr} = 1, q = i|\theta^g) \\
&= \sum_{j=1}^{k} \sum_{r=1}^{n_j} p(D_j, q = i|\theta^g) p(z_{jr} = 1|D_j, q = i, \theta^g) \\
&= \sum_{j=1}^{k} \sum_{r=1}^{n_j} p(D_j, q = i|\theta^g) p(z_{jr} = 1|D_j, q = i, \theta^g) \\
&= \sum_{j=1}^{k} \sum_{r=1}^{n_j} p(D_j, q = i|\theta^g) p(z_{jr} = 1|q = i, \theta^g) \\
&= \sum_{j=1}^{k} \sum_{r=1}^{n_j} p(D_j, q = i|\theta^g) \frac{\beta_i^g p(o_{jr}|\mu_{j1})}{\beta_i^g p(o_{jr}|\mu_{j1}) + (1 - \beta_i^g) p(o_{jr}|\mu_{j2})}
\end{aligned}$$

Therefore: $\beta_i^{g+1} = \frac{\sum_{j=1}^{k} p(D_j, q = i|\theta^g) \sum_{r=1}^{n_j} \frac{\beta_i^g p(o_{jr}|\mu_{j1})}{\beta_i^g p(o_{jr}|\mu_{j1}) + (1 - \beta_i^g) p(o_{jr}|\mu_{j2})} + (\nu_{i1} - 1) \sum_{j=1}^{k} p(D_j|\theta^g)}{\sum_{j=1}^{k} n_j p(D_j, q = i|\theta^g) + (\nu_{i1} + \nu_{i2} - 2) \sum_{j=1}^{k} p(D_j|\theta^g)}$

Also, we notice that:

$$p(q = i|D_j, \theta^g) = \frac{\alpha_i^g e^{-\theta_i \lambda^g} \frac{(\theta_i \lambda^g)^{f_j}}{f_j!} (\beta_i^g)^{n_{j1}} (1-\beta_i^g)^{n_{j2}}}{\sum_{i=0}^{m} \alpha_i e^{-\theta_i \lambda} \frac{(\theta_i \lambda)^{f_j}}{f_j!} (\beta_i^g)^{n_{j1}} (1-\beta_i^g)^{n_{j2}}}$$

As explained above, n_{j1} determines the number of mate pairs which are mapped to the reference genome with the clone library insertion size distribution. Also, n_{j2} determines the number of mate pairs which are mapped with a shifted insertion size distribution, compared to the clone library. Here, n_{j1} and n_{j2} are estimated based on the insertion sizes i.e. o_{jr}, j=1, 2, …, k and $r = 1,2, \dots, n_j$, which are observed in the mate pair reads from j[th] sample, after mapping to the reference genome.

Indeed, o_{jr} comes from the clone library insertion size distribution with the following probability:

$$p(z_{jr} = 1) = \sum_{i=0}^{m} \alpha_i^g \frac{\beta_i^g p(o_{jr}|\mu_{j1})}{\beta_i^g p(o_{jr}|\mu_{j1}) + (1-\beta_i^g) p(o_{jr}|\mu_{j2})}$$

In which, $p(o_{jr}|\mu_{j1})$ is the empirical distribution of the insertion sizes in the clone library. Also, $p(o_{jr}|\mu_{j2})$ is the shifted empirical distribution of the insertion sizes, where the shift is equal to $|\mu_{j2} - \mu_{j1}|$. Consequently, in the gth iteration of the EM algorithm, the expected value of n_{j1} and n_{j2} are given as follows:

$$E(n_{j1}) = \sum_{r=1}^{n_j} p(z_{jr} = 1)$$
$$E(n_{j2}) = \sum_{r=1}^{n_j} [1 - p(z_{jr} = 1)]$$

Generally, based on above descriptions, the EM algorithm takes the following steps for parameter re-estimation, in each iteration:

EM iterations for parameter re-estimation:

0- Initialize $\{\mu_{j1}, \mu_{j2}\}$ for j=1,2,…,k and all other parameters

1- Calculate $p(z_{jr} = 1)$, which is the probability of o_{jr} coming from the insertion size distribution of the clone library.

$$p(z_{jr} = 1) = \sum_{i=0}^{m} \alpha_i^g \frac{\beta_i^g p(o_{jr}|\mu_{j1})}{\beta_i^g p(o_{jr}|\mu_{j1}) + (1-\beta_i^g) p(o_{jr}|\mu_{j2})}$$

2- Compute the expected value of n_{j1} and n_{j2} based on above probabilities:

$$E(n_{j1}) = \sum_{r=1}^{n_j} p(z_{jr} = 1)$$
$$E(n_{j2}) = \sum_{r=1}^{n_j} [1 - p(z_{jr} = 1)]$$

3- Update $\boldsymbol{\alpha} = (\alpha_0, \alpha_1, \dots, \alpha_m)$, $\boldsymbol{\beta} = (\beta_0, \beta_1, \dots, \beta_m)$ and λ

4- In the jth sample, update $\{\mu_{j1}, \mu_{j2}\}$ based on the following EM formula:

$$\mu_{j1} = \frac{\sum_{r=1}^{n_j} o_{jr} P(z_{jr}=1|o_{jr}, \mu_{j1})}{\sum_{r=1}^{n_j} P(z_{jr}=1|o_{jr}, \mu_{j1})}$$

$$\mu_{j2} = \frac{\sum_{r=1}^{n_j} o_{jr} P(z_{jr} = 0|o_{jr}, \mu_{j2})}{\sum_{r=1}^{n_j} P(z_{jr} = 0|o_{jr}, \mu_{j2})}$$

5- Repeat steps 1 to 4, until reaching convergence

Table S.1: Number of deletion and duplication calls which are made by MSeq-CNV in the high-coverage sequencing data of NA12891, NA12892, NA12878, NA19238, NA19239, NA19240, for 22 autosomal and X chromosomes.

	**Deletions**						**Duplications**					
	NA12891	NA12892	NA12878	NA19238	NA19239	NA19240	NA12891	NA12892	NA12878	NA19238	NA19239	NA19240
Chr. 1	6,745	7,096	6,975	9,503	6,527	6,002	17,219	17,335	17,029	15,865	15,368	15,639
Chr. 2	6,586	6,908	6,911	9,575	6,472	6,150	8,397	8,375	8,290	9,055	9,850	11,025
Chr. 3	5,062	5,101	5,329	7,265	5,037	4,810	6,899	6,505	7,079	7,354	7,951	8,697
Chr. 4	1,617	5,531	5,606	7,199	5,420	5,071	16,048	6,396	6,710	6,973	7,443	8,433
Chr. 5	5,262	5,566	5,590	7,072	5,296	4,992	7,124	7,207	7,415	7,583	7,959	8,974
Chr. 6	4,711	4,777	4,850	6,768	4,710	4,476	5,694	5,239	5,748	5,919	6,662	7,559
Chr. 7	5,667	5,849	5,855	7,764	5,316	4,952	7,684	8,164	8,029	8,026	7,595	8,123
Chr. 8	3,745	3,802	3,653	5,339	3,845	3,679	5,226	4,873	5,732	5,369	5,741	6,807
Chr. 9	4,120	4,280	4,424	4,524	3,945	3,651	12,857	12,813	12,814	12,504	12,618	12,612
Chr. 10	3,834	3,769	3,986	5,116	3,759	3,541	5,145	5,232	4,840	5,193	5,610	6,268
Chr. 11	4,141	4,310	4,497	6,269	4,119	4,016	5,863	6,603	5,706	6,046	6,145	6,628
Chr. 12	3,610	3,789	3,828	5,876	3,555	3,271	4,669	5,006	4,970	5,199	5,144	5,829
Chr. 13	1,851	1,911	1,939	2,552	1,925	1,628	8,890	9,087	8,867	8,548	8,393	8,771
Chr. 14	2,202	2,281	2,342	3,019	2,196	1,967	9,103	9,170	9,029	8,677	8,488	8,675
Chr. 15	2,434	2,465	2,485	2,996	2,446	2,287	9,858	9,730	9,804	9,245	9,433	9,264
Chr. 16	2,842	3,051	3,007	4,156	2,682	2,490	6,859	6,967	6,946	6,472	6,624	6,611
Chr. 17	2,428	2,681	2,709	4,151	2,309	2,135	5,372	5,358	5,254	5,028	5,031	4,912
Chr. 18	1,642	1,708	1,757	2,354	1,620	1,530	3,720	3,144	3,243	3,301	3,904	3,910
Chr. 19	1,479	1,430	1,496	3,066	1,528	1,271	3,976	3,832	3,871	3,700	4,194	4,241
Chr. 20	1,239	1,312	1,409	2,403	1,262	1,163	3,362	3,574	3,301	3,413	3,227	3,569
Chr. 21	726	754	753	997	689	623	4,557	4,445	4,379	4,367	4,501	4,316
Chr. 22	1,061	1,206	1,279	1,467	1,041	1,073	5,213	4,644	4,921	4,857	5,755	5,453
Chr. X	6,205	5,919	5,948	7,540	5,989	5,391	6,460	9,694	8,557	8,362	6,905	8,784
Total	79,209	85,496	86,628	116,971	81,688	76,169	170,195	163,393	162,534	161,056	164,541	175,100

Table S.2: Total size of deletion and duplication calls (in Mega bp) detected by MSeq-CNV in the high-coverage sequencing data of NA12891, NA12892, NA12878, NA19238, NA19239, NA19240, for 22 autosomal and X chromosomes.

	Deletions						Duplications					
	NA12891	NA12892	NA12878	NA19238	NA19239	NA19240	NA12891	NA12892	NA12878	NA19238	NA19239	NA19240
Chr. 1	12.120	12.277	12.308	16.929	12.649	11.692	29.616	26.924	28.903	26.360	30.557	33.452
Chr. 2	12.367	12.793	12.877	17.314	12.611	11.942	11.564	10.575	11.148	12.703	16.266	22.117
Chr. 3	8.769	8.841	9.111	12.502	9.238	8.703	9.532	8.149	9.561	10.138	13.419	17.254
Chr. 4	2.732	9.684	9.920	13.115	10.250	9.546	29.199	8.106	9.300	9.895	12.159	15.245
Chr. 5	9.437	10.023	10.183	13.042	10.109	9.589	10.227	9.292	10.489	11.098	14.051	17.608
Chr. 6	8.101	8.102	8.402	11.611	8.624	8.230	7.700	6.523	7.667	8.069	10.716	14.222
Chr. 7	10.876	10.929	11.186	14.848	10.790	10.092	11.413	11.365	12.114	12.183	13.164	15.674
Chr. 8	6.574	6.705	6.514	9.411	7.156	6.857	7.296	6.124	7.632	7.571	9.322	13.229
Chr. 9	8.066	8.061	8.609	8.764	8.046	7.630	24.664	22.238	23.315	21.489	25.576	23.535
Chr. 10	6.913	6.823	7.246	9.074	7.156	6.790	7.160	6.747	6.595	7.342	9.289	12.700
Chr. 11	7.218	7.538	7.893	11.213	7.889	7.761	8.606	9.161	8.254	9.194	10.767	14.865
Chr. 12	6.053	6.200	6.407	9.961	6.426	5.854	6.442	6.414	6.837	7.382	8.348	11.615
Chr. 13	3.195	3.252	3.293	4.445	3.469	2.940	15.899	14.458	15.328	14.382	16.479	15.803
Chr. 14	3.729	4.025	4.129	5.393	4.238	3.741	17.268	15.266	15.917	14.587	16.977	16.104
Chr. 15	4.548	4.549	4.609	5.635	4.807	4.582	18.921	15.780	17.269	15.482	19.477	17.525
Chr. 16	5.472	5.715	5.759	7.872	5.448	5.004	12.432	11.570	12.510	11.088	13.398	14.177
Chr. 17	4.572	4.852	4.805	7.217	4.450	4.373	8.757	8.166	8.713	8.151	9.195	10.788
Chr. 18	3.034	2.992	3.146	4.061	3.032	2.861	5.487	4.029	4.487	4.715	7.044	7.405
Chr. 19	2.256	2.146	2.264	4.582	2.578	2.181	6.277	5.239	5.958	5.327	7.684	8.256
Chr. 20	2.012	2.059	2.198	3.973	2.176	2.069	4.874	5.036	4.850	5.542	5.169	7.076
Chr. 21	1.330	1.335	1.382	1.839	1.337	1.239	8.019	7.039	7.204	6.992	8.047	7.436
Chr. 22	2.051	2.145	2.356	2.711	2.057	2.111	8.103	6.206	7.343	6.886	9.628	10.050
Chr. X	12.022	10.662	10.868	14.179	11.990	10.557	8.391	14.373	13.345	13.353	10.146	16.426
Total	143.442	151.703	155.460	209.685	156.520	146.339	277.842	238.777	254.734	249.925	296.874	342.556

Table S.3: Total number and size of deletion and duplication calls (in Mega bp) detected by MSeq-CNV in the low-coverage sequencing data of NA12761, NA12762, for 22 autosomal and X chromosomes.

	Deletions				Duplications				Overall			
	By Calls		By size (in Mega bp)		By Calls		By size (in Mega bp)		By Calls		By size (in Mega bp)	
	NA12761	NA12762	NA12761	NA12762	NA12761	NA12762	NA12761	NA12762	NA12761	NA12762	NA12761	NA12762
Chr. 1	4,506	4,278	7.156	6.944	7,691	5,771	8.728	6.591	12,197	10,049	15.883	13.535
Chr. 2	5,992	4,207	9.188	6.514	4,417	2,931	4.980	3.135	10,409	7,138	14.168	9.648
Chr. 3	2,750	8	3.963	0.014	4,164	156	4.609	0.688	6,914	164	8.571	0.702
Chr. 4	3,274	3,115	4.809	4.587	3,783	1,278	4.363	1.390	7,057	4,393	9.172	5.977
Chr. 5	3,399	3,281	5.245	5.071	4,021	2,040	4.517	2.193	7,420	5,321	9.762	7.263
Chr. 6	2,690	2,627	4.069	4.021	3,300	2,171	3.648	2.449	5,990	4,798	7.716	6.470
Chr. 7	3,660	3,567	5.688	5.762	3,771	1,700	4.291	1.833	7,431	5,267	9.978	7.595
Chr. 8	2,554	2,441	3.894	3.739	3,089	1,755	3.365	1.886	5,643	4,196	7.258	5.625
Chr. 9	3,379	3,559	6.151	6.641	7,862	5,067	9.334	5.771	11,241	8,626	15.485	12.412
Chr. 10	3,334	3,134	5.496	5.347	2,797	1,317	3.113	1.452	6,131	4,451	8.609	6.799
Chr. 11	3,184	3,031	4.714	4.600	3,755	1,377	4.342	1.484	6,939	4,408	9.056	6.084
Chr. 12	2,645	550	3.754	0.812	2,469	12,256	2.755	15.998	5,114	12,806	6.509	16.810
Chr. 13	906	848	1.268	1.198	4,363	3,562	4.994	4.052	5,269	4,410	6.261	5.249
Chr. 14	1,553	1,524	2.342	2.327	4,969	3,163	5.740	3.485	6,522	4,687	8.082	5.812
Chr. 15	2,087	1,607	3.596	2.824	6,499	4,880	7.754	5.786	8,586	6,487	11.350	8.609
Chr. 16	2,064	778	3.484	1.739	3,092	7,768	3.625	18.341	5,156	8,546	7.109	20.080
Chr. 17	1,974	1,473	3.280	2.499	2,729	1,894	3.091	2.086	4,703	3,367	6.371	4.585
Chr. 18	1,094	1,070	1.578	1.563	1,658	932	1.826	0.989	2,752	2,002	3.404	2.552
Chr. 19	1,050	711	1.501	1.074	2,235	1,656	2.551	1.822	3,285	2,367	4.052	2.896
Chr. 20	804	750	1.139	1.088	1,741	873	1.994	0.950	2,545	1,623	3.133	2.038
Chr. 21	364	363	0.545	0.536	3,237	2,387	4.011	2.810	3,601	2,750	4.556	3.346
Chr. 22	760	775	1.287	1.332	4,982	3,849	6.145	4.537	5,742	4,624	7.431	5.869
Chr. X	2,392	3,844	4.534	6.380	2,423	2,750	3.050	3.007	4,815	6,594	7.584	9.387
Total	56,415	47,541	88.675	76.609	89,047	71,533	102.820	92.730	145,462	119,074	191.495	169.339

Table S.4: Number of CNV calls which are made by MSeq-CNV in the genome of six individuals from the Simons Genome Diversity Project i.e. LP6005592-DNA_H03 (USA), LP6005442-DNA_E07 (Taiwan), LP6005443-DNA_G05 (Taiwan), LP6005519-DNA_A04 (India), LP6005519-DNA_A05 (India), and LP6005592-DNA_D01 (Finland).

	**Deletions**						**Duplications**					
	**H03**	**E07**	**G05**	**A04**	**A05**	**D01**	**H03**	**E07**	**G05**	**A04**	**A05**	**D01**
Chr. 1	2,730	4,528	3,535	7,893	8,155	4,167	15,066	12,048	13,622	15,748	18,360	14,621
Chr. 2	3,249	5,575	4,656	11,960	20,901	6,098	1,673	1,346	1,343	1,269	1,674	1,272
Chr. 3	1,578	3,857	2,421	7,775	12,849	2,850	689	535	542	428	622	456
Chr. 4	2,268	4,040	3,342	10,033	20,786	3,290	683	499	673	383	488	425
Chr. 5	2,231	3,494	2,640	6,584	17,391	3,193	1,066	736	996	759	1,133	774
Chr. 6	1,321	3,837	3,403	12,621	8,814	2,929	576	548	567	389	628	464
Chr. 7	2,679	4,037	3,837	9,148	10,892	3,471	1,573	1,212	1,572	1,511	2,087	1,649
Chr. 8	1,726	4,631	2,256	5,886	11,662	3,328	900	729	859	587	817	763
Chr. 9	3,208	3,345	3,335	3,852	4,548	3,686	24,014	24,287	24,019	23,042	22,481	22,801
Chr. 10	1,835	5,692	3,000	8,036	10,439	2,318	1,965	1,614	1,744	1,502	1,952	1,422
Chr. 11	1,319	2,352	2,907	8,668	8,639	3,176	1,128	771	1,033	1,025	1,519	1,029
Chr. 12	1,434	5,902	1,785	5,654	16,529	1,941	664	571	691	508	787	582
Chr. 13	494	744	715	1,383	1,510	665	15,758	15,466	15,908	16,900	16,747	16,648
Chr. 14	818	1,036	925	1,046	2,077	812	16,333	16,412	17,032	16,576	15,786	16,078
Chr. 15	1,797	1,829	1,751	2,154	4,471	1,726	17,477	18,265	17,472	16,795	16,356	17,165
Chr. 16	1,764	2,152	2,215	4,390	4,076	1,905	6,582	6,009	6,087	6,540	5,811	6,691
Chr. 17	1,119	1,346	2,063	5,914	5,826	1,966	2,782	2,399	2,436	3,198	4,178	3,029
Chr. 18	612	1,063	1,183	3,721	4,907	822	1,582	1,458	1,460	1,525	1,935	1,480
Chr. 19	747	1,437	1,237	3,748	5,547	1,207	1,672	1,350	1,647	2,095	2,622	1,969
Chr. 20	494	1,684	645	1,926	4,075	958	1,778	1,533	1,788	2,308	2,658	1,950
Chr. 21	442	428	477	595	814	517	7,180	7,494	7,023	6,039	5,979	6,334
Chr. 22	667	641	743	733	957	707	9,346	9,550	9,331	8,907	9,750	8,997
Chr. X	3,640	6,464	3,763	7,817	18,839	4,759	2,751	2,489	2,760	2,834	2,835	2,747
Total	38,172	70,114	52,834	131,537	204,704	56,491	133,238	127,321	130,605	130,868	137,205	129,346

Table S.5: Total size of deletion and duplication calls (in Mega bp) detected by MSeq-CNV in the genome of six individuals from the Simons Genome Diversity Project i.e. LP6005592-DNA_H03 (USA), LP6005442-DNA_E07 (Taiwan), LP6005443-DNA_G05 (Taiwan), LP6005519-DNA_A04 (India), LP6005519-DNA_A05 (India), and LP6005592-DNA_D01 (Finland).

	Deletions						Duplications					
	**H03**	**E07**	**G05**	**A04**	**A05**	**D01**	**H03**	**E07**	**G05**	**A04**	**A05**	**D01**
Chr. 1	5.068	7.217	6.091	11.692	12.062	6.879	19.773	14.758	17.446	20.484	24.850	18.754
Chr. 2	5.518	8.222	7.298	16.796	29.664	9.075	1.892	1.500	1.511	1.557	1.981	1.414
Chr. 3	2.515	5.169	3.475	10.192	17.226	4.038	0.759	0.592	0.608	0.463	0.681	0.493
Chr. 4	3.528	5.557	4.766	13.432	29.334	4.782	0.756	0.545	0.758	0.471	0.529	0.484
Chr. 5	3.772	5.206	4.233	9.336	24.549	4.849	1.181	0.797	1.115	0.815	1.261	0.880
Chr. 6	2.070	4.988	4.541	16.637	11.608	4.019	0.715	0.742	0.718	0.532	0.779	0.604
Chr. 7	4.587	6.138	6.136	13.523	16.313	5.739	1.785	1.350	1.781	1.678	2.348	1.855
Chr. 8	2.937	6.368	3.586	8.223	16.552	4.952	1.040	0.830	0.989	0.665	0.909	0.869
Chr. 9	6.628	6.647	6.902	7.900	8.555	7.515	48.959	46.081	48.828	51.643	51.853	49.915
Chr. 10	3.232	7.992	4.740	11.348	14.911	3.958	2.331	1.860	2.068	1.727	2.251	1.637
Chr. 11	2.219	3.378	4.043	11.975	12.287	4.481	1.249	0.961	1.177	1.127	1.680	1.110
Chr. 12	2.097	7.503	2.500	7.416	24.057	2.704	0.728	0.611	0.756	0.548	0.859	0.640
Chr. 13	0.752	1.098	1.022	1.842	2.059	0.992	24.281	22.253	24.525	26.800	27.047	26.122
Chr. 14	1.336	1.704	1.618	1.660	2.964	1.309	27.421	26.540	29.490	30.191	28.869	28.602
Chr. 15	3.311	3.236	3.211	3.932	6.913	3.009	39.518	38.403	38.594	40.581	38.121	37.762
Chr. 16	3.234	3.838	3.958	6.851	6.526	3.536	9.202	8.012	8.331	9.306	8.306	9.674
Chr. 17	1.876	2.163	3.035	8.330	8.218	2.907	3.489	2.878	2.988	4.066	5.592	3.813
Chr. 18	0.983	1.457	1.613	4.859	6.538	1.169	1.804	1.619	1.668	1.710	2.193	1.662
Chr. 19	1.246	2.074	1.771	5.118	7.857	1.764	2.135	1.735	2.052	2.765	3.730	2.529
Chr. 20	0.738	2.089	0.900	2.586	5.463	1.305	2.107	1.763	2.113	2.810	3.332	2.349
Chr. 21	0.811	0.794	0.851	1.026	1.252	0.931	19.538	19.512	19.502	17.596	17.336	17.477
Chr. 22	1.261	1.209	1.438	1.462	1.713	1.324	23.300	23.862	23.175	23.150	23.969	23.277
Chr. X	5.564	8.855	5.610	10.690	26.475	6.901	3.830	3.546	3.878	3.911	3.876	3.782
Total	65.276	102.897	83.333	186.819	293.091	88.131	237.788	220.741	234.064	244.590	252.344	235.698
